# MIR100HG: a credible prognostic biomarker and an oncogenic lncRNA in gastric cancer

**DOI:** 10.1042/BSR20190171

**Published:** 2019-04-05

**Authors:** Jun Li, Qingfeng Xu, Wen Wang, Shaojun Sun

**Affiliations:** 1 Department of Clinical Laboratory, Jining NO. 1 People’s Hospital; Affiliated Jining NO. 1 People’s Hospital of Jining Medical University, Jining Medical University, Jining 272011, Shandong, China; 2 Department of Hematology-Oncology, People’s Hospital of Gaotang County Affiliated to Jining Medical University, Liaocheng 252800, Shandong, China; 3 Department of Clinical Laboratory, People’s Hospital of Binzhou City, Binzhou 256600, Shandong, China; 4 Department of Clinical Laboratory, Brain Hospital of Liaocheng City, People’s Hospital of Liaocheng City, Liaocheng 252000, Shandong, China

**Keywords:** biomarkers, gastric cancer, large intervening non-coding RNA, MIR100HG

## Abstract

The MIR100HG expression was observed to be up-regulated or down-regulated in human cancer tissues depending on tumor types. However, there was no report about the role of MIR100HG in gastric cancer. In our study, we first found levels of MIR100HG expression were increased in gastric cancer cell lines and tissue samples compared with normal gastric epithelial cell line and adjacent normal gastric mucosa tissue samples, respectively. Moreover, high MIR100HG expression was positively associated with clinical stage, tumor invasion, lymph node metastasis, and distant metastasis in gastric cancer patients. Survival analysis showed MIR100HG expression was negative correlated with clinical outcome in gastric cancer patients from The Cancer Genome Atlas (TCGA) database or our study, and high MIR100HG expression served as an independent poor prognostic factor for gastric cancer patient’s overall survival. The study *in vitro* suggested down-regulation of MIR100HG expression inhibits cell proliferation, migration, and invasion in gastric cancer. In conclusion, MIR100HG is a credible prognostic biomarker and functions as an oncogenic lncRNA in gastric cancer.

## Introduction

Gastric cancer is one of most prevalent human cancers originated from digestive system, and the second leading cause of cancer-related death worldwide at 2018 [2]. Up to now, surgery remains the most effective therapy for gastric cancer patients with early stage [15,28]. Owing to lack of specific symptoms in the early stage, most patients with gastric cancer is advanced stage at diagnosis [9,12]. Although there were great advances in chemotherapy, radiotherapy, and novel targetted therapy recent decades, the clinical outcome is still unsatisfactory in gastric cancer patients with advanced stage [19,20]. Thus, it is important to explore valuable biomarkers predicting clinical progression and prognosis in gastric cancer patients, which provide substantial theoretical basis for identifying high risk patients and making treatment strategies.

lncRNAs, one member of non-coding RNAs family, are greater than 200 nts in length and have limited protein-coding ability [5,6]. Growing evidence has suggested that several lncRNAs served as critical roles in gastric cancer carcinogenesis, such as ANRIL [25][10], THOR [17], ENST00000434223 [21], MEG3 [4], AK096174, [27] and so on. LncRNA mir-100-let-7a-2-mir-125b-1 cluster host gene (MIR100HG) originally identified in a human transcriptome analysis [8] and described as a crucial function in neuronal differentiation of human neural stem cells [13]. Whereafter, MIR100HG was been showed to be dysregulated and function as tumor promoter or suppressor in human cancers including lung cancer [26], breast cancer [3,11,22], colorectal cancer [10], bladder cancer [1,23], cervical cancer [16], osteosarcoma [18], pancreatic ductal adenocarcinoma [14] head and neck squamous cell carcinoma, [24] and acute megakaryoblastic leukemia [7]. Up to now, the expression status of MIR100HG in gastric cancer is still unknown. In our study, we investigated the clinical and prognostic value of MIR100HG in gastric cancer patients through analyzing the relationship between MIR100HG and clinicopathological features. Moreover, we conducted loss-of-function study to preliminarily explore the effect of MIR100HG on gastric cancer cell proliferation, migration, and invasion.

## Materials and methods

### Tissue samples

The study was approved by the Ethics Committee of Jining No.1 People’s Hospital, People’s Hospital of Gaotang County, People’s Hospital of Binzhou City and People’s Hospital of Liaocheng City. All participators signed the informed consent and were aware of the study detail. Total 122 fresh frozen gastric cancer tissue samples and 40 adjacent normal gastric mucosa tissue samples were collected from Jining No.1 People’s Hospital, People’s Hospital of Gaotang County, People’s Hospital of Binzhou City and People’s Hospital of Liaocheng City. All tissue specimens were frozen in liquid nitrogen and preserved in -80°C, and the pathologic diagnosis of each tissue specimen has been confirmed by at least two pathologists. Prior to surgery or biopsy, all patients did not receive any antitumor therapy. Clinical tumor stage was evaluated according to the AJCC classification criteria.

TCGA gastric cancer cohort was analyzed in the present study. MIR100HG expression data and survival data of 379 gastric cancer cases were included in survival analysis.

### RNA extraction and qRT-PCR

Total RNAs from tissue samples and cell lines were extracted by TRIzol reagent (Invitrogen, Carlsbad, CA, U.S.A.). According to the manufacturer’s guide, PrimeScript RT Master Mix (Takara, Dalian, China) was utilized for reverse transcription. Then, quantitative real-time PCR (qRT-PCR) analysis was conducted by One Step TB Green PrimeScript RT-PCR Kit (Takara, Dalian, China) at an ABI 7500 real-time PCR System (Applied Biosystems, Foster City, CA, U.S.A.). The primer sequence details were as follows: MIR100HG forward, 5’-GGCGACATCAGACAGACAGA-3’ and reverse, 5’-AGGACCAGCTGAAAGGAACA-3’; β-actin forward, 5’-AAAGACCTGTACGCCAACAC-3’ and reverse, 5’-GTCATACTCCTGCTTGCTGAT-3’. β-actin was taken as an internal control. MIR100HG and β-actin were amplified in different wells and run in triplicate.

### Cell lines and cell transfection

Human gastric epithelial cell line (GES-1) and human gastric cancer cell lines (MGC-803, SGC7901, BGC-823, AGS) were cultured in PRMI-1640 medium (HyClone, Logan, UT, U.S.A.) supplement with 10% fetal bovine serum (FBS; Invitrogen, Carlsbad, CA, U.S.A.) at a humidified incubator with 5% CO_2_ and 37°C.

Specific siRNA targetting MIR100HG (si-MIR100HG) and scrambled negative control (si-NC) were obtained by GenePharma (Shanghai, China) and transfected into cultured gastric cancer cell lines through Lipofectamine RNAiMAX Transfection Reagent (Life Technologies, Thermo Fisher Scientific Inc., Waltham, MA, U.S.A.).

### Cell counting kit-8 assay

Cell proliferative ability was assessed by cell counting kit-8 (CCK-8) assay (Dojindo Molecular Technologies, Rockville, MD, U.S.A.). Gastric cancer cells were transfected with siRNAs for 24 h and plated into 96-well plates. After further incubation for 1, 3 and 5 days respectively, 10 μl of CCK-8 solution was added into corresponding wells for 2 h. Finally, the OD (optical density) value of each well was measured by a microplate reader under the wavelength of 450 nm. The assay was conducted in duplicate in three independent experiments.

### Migration and invasion assay

The migration assay was conducted in 24-well Transwell chambers with 8‐μm pore size polycarbonate membranes (BD Biosciences, Franklin Lakes, NJ, U.S.A.), but invasion assay conducted in Transwell chambers with Matrigel (BD Biosciences, Franklin Lakes, NJ, U.S.A.) coated membranes. Briefly, transfected gastric cancer cells (5 × 10^4^ cells/well) were seeded with serum-free medium into the upper chambers, and medium with 15% FBS was added into the bottom chambers. After 24-h incubation, gastric cancer cells at the upper surface of membranes were removed, and gastric cancer cells penetrating to the lower side of membranes were fixed with methanol for 30 min and stained with 0.1% crystal violet solution for 20 min. Finally, the cell numbers were counted in five randomly chosen microscopic fields per membrane. These experiments were repeated three times.

### Statistical analysis

The data were analyzed with the SPSS 17.0 (SPSS, Inc., Chicago, IL, U.S.A.). The correlation between MIR100HG expression and clinicopathological factors of gastric cancer patients was assessed by chi-square test. The statistic difference between two groups was estimated by the Student’s *t*-test. The Kaplan–Meier method and log-rank test were used to evaluate overall survival of gastric cancer patients with different MIR100HG expression. Univariate and multivariate Cox proportional hazard regression models were applied to analyze independent prognostic factors for overall survival in gastric cancer patients. All *P*-values were two-tailed, and *P*-value < 0.05 was considered as statistically significant. Table 1.

**Table 1:** Relationships between MIR100HG expression and clinicopathological parameters in gastric cancer

Parameters	n	High expression (%)	Low expression (%)	*P*-value
Gender				
Female	46	25 (54.3)	21 (45.7)	0.455
Male	76	36 (47.4)	40 (52.6)	
Age (y)				
<50	47	21 (44.7)	26 (55.3)	0.352
≥50	75	40 (53.3)	35 (46.7)	
Histological type				
Differentiated	71	32 (45.1)	39 (54.9)	0.199
Undifferentiated	51	29 (56.9)	22 (43.1)	
Clinical stage				
I–II	51	14 (27.5)	37 (72.5)	<0.001
III–IV	71	47 (66.2)	24 (33.8)	
Tumor depth				
T1–T2	62	22 (35.5)	40 (64.5)	0.001
T3–T4	60	39 (65.0)	21 (35.0)	
Lymph node metastasis				
N0–N1	59	20 (33.9)	39 (66.1)	0.001
N2–N3	63	41 (65.1)	22 (34.9)	
Distant metastasis				
M0	110	50 (45.5)	60 (54.5)	0.002
M1	12	11 (91.7)	1 (8.3)	
HP infection				
Absent	83	39 (47.0)	44 (53.0)	0.332
Present	39	22 (56.4)	17 (43.6)	

## Results

### MIR100HG was up-regulated in gastric cancer

**Figure 1:**
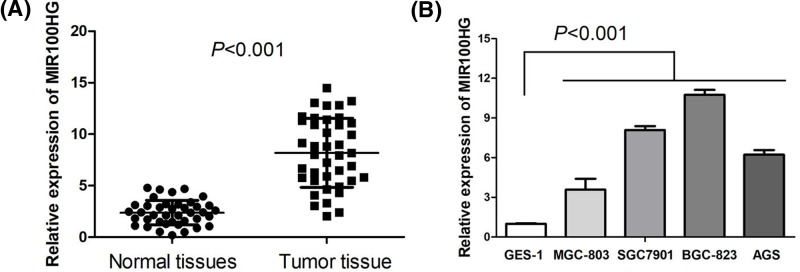
MIR100HG is up-regulated in gastric cancer (**A**) MIR100HG expression was markedly increased in gastric cancer tissue samples rather than that in adjacent normal normal gastric mucosa tissue samples. (**B**) MIR100HG expression levels were obviously elevated in human gastric cancer cell lines compared with human gastric epithelial cell line.

First, qRT-PCR was performed to measure the expression pattern of MIR100HG in gastric cancer tissue samples and adjacent normal normal gastric mucosa tissue samples. The result suggested MIR100HG expression was markedly increased in gastric cancer tissue samples rather than that in adjacent normal normal gastric mucosa tissue samples (*P*<0.001, Figure 1A). Furthermore, we detected the expression of MIR100HG in a human gastric epithelial cell line (GES-1) and four human gastric cancer cell lines (MGC-803, SGC7901, BGC-823, AGS) through qRT-PCR. We observed that MIR100HG expression levels were obviously elevated in human gastric cancer cell lines compared with human gastric epithelial cell line (*P*<0.001, Figure 1B).

### MIR100HG is associated with clinical progression in gastric cancer

To further estimate the clinical value of MIR100HG in gastric cancer, all gastric cancer tissue samples were divided into low MIR100HG expression group and high MIR100HG expression group based the median value of MIR100HG expression, and the association between MIR100HG expression and clinicopathological features of gastric cancer patient’s was estimated by chi-square test. In our result, we found high MIR100HG expression was positively associated with clinical stage (*P*<0.001), tumor invasion (*P*=0.001), lymph node metastasis (*P*=0.001), and distant metastasis (*P*=0.002), but not with gender (*P*=0.455), age (*P*=0.352), histological grade (*P*=0.199), and HP infection (*P*=0.332).

### MIR100HG is associated with poor clinical outcome in gastric cancer

**Figure 2:**
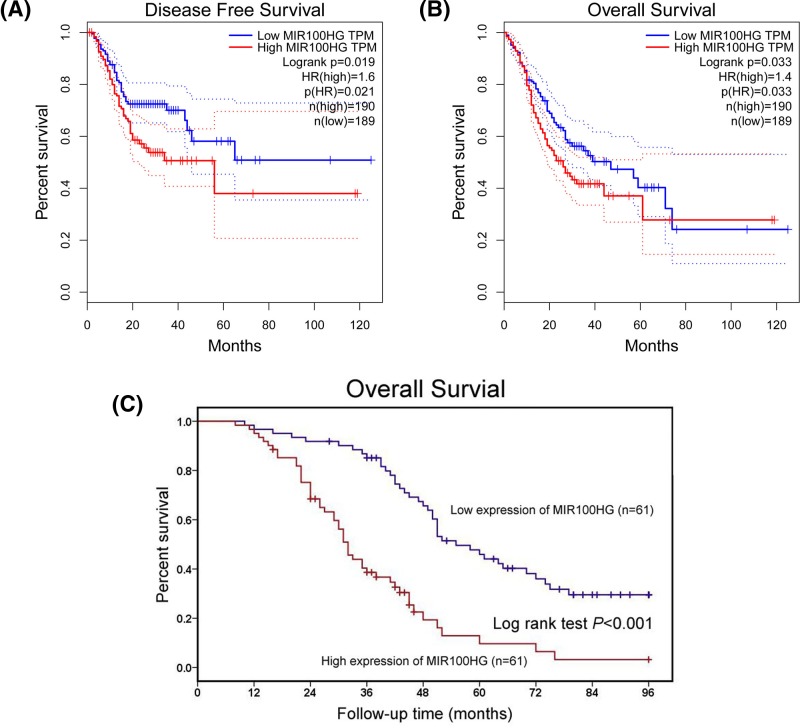
MIR100HG is associated with poor clinical outcome in gastric cancer (**A**) The relationship between MIR100HG expression and disease free survival and overall survival in TCGA gastric cancer cohort. (**B**) The relationship between MIR100HG expression and overall survival in TCGA gastric cancer cohort. (**C**) The relationship between MIR100HG expression and overall survival in our study gastric cancer cohort.

For exploring the prognostic value of MIR100HG in gastric cancer patients, Kaplan–Meier method and log-rank test were executed to assess the correlation between MIR100HG expression and clinical outcome of gastric cancer patients at TCGA database and our study. As shown in Figure 2A,B, we found that MIR100HG expression is negatively associated with disease free survival (*P*=0.019) and overall survival (*P*=0.033) in TCGA gastric cancer cohort. Similarly, we also found high MIR100HG expression of predicted short overall survival time in gastric cancer patients from our study (*P*<0.001, Figure 2C). According to univariate and multivariate Cox proportional hazard regression models, high MIR100HG expression was identified as an independent poor prognostic factor for gastric cancer patient’s overall survival (*P*=0.022, Table 2).

**Table 2:** Univariate and multivariate Cox regression analyses of overall survival in gastric cancer

Parameter	Univariate analysis			Multivariate analysis		
	Hazard ratio	95% CI	*P*-value	Hazard ratio	95% CI	*P*-value
Gender						
(Female vs. male)	0.969	0.629–1.493	0.886			
Age						
(<50 vs. ≥50)	1.158	0.754–1.778	0.504			
Histological grade						
(Differentiated vs.undifferentiated)	1.131	0.741–1.728	0.568			
Clinical stage						
(I-II vs. III-IV)	3.959	2.387–6.565	<0.001	1.913	0.636–5.759	0.248
Tumor depth						
(T1-T2 vs. T3-T4)	1.958	1.277–3.001	0.002	1.588	0.992–2.544	0.054
Lymph node metastasis						
(N0-N1 vs. N2-N3)	3.628	2.235–5.890	<0.001	1.379	0.489–3.891	0.544
Distant metastasis						
(M0 vs. M1)	6.149	3.191–11.850	<0.001	3.214	1.568–6.588	0.001
HP infection						
(Absent vs. present)	1.103	0.712–1.708	0.679			
MIR100HG expression						
(Low vs. high)	3.265	2.089–5.104	<0.001	1.851	1.093–3.135	0.022

### Down-regulation of MIR100HG expression inhibits cell proliferation, migration, and invasion in gastric cancer

**Figure 3:**
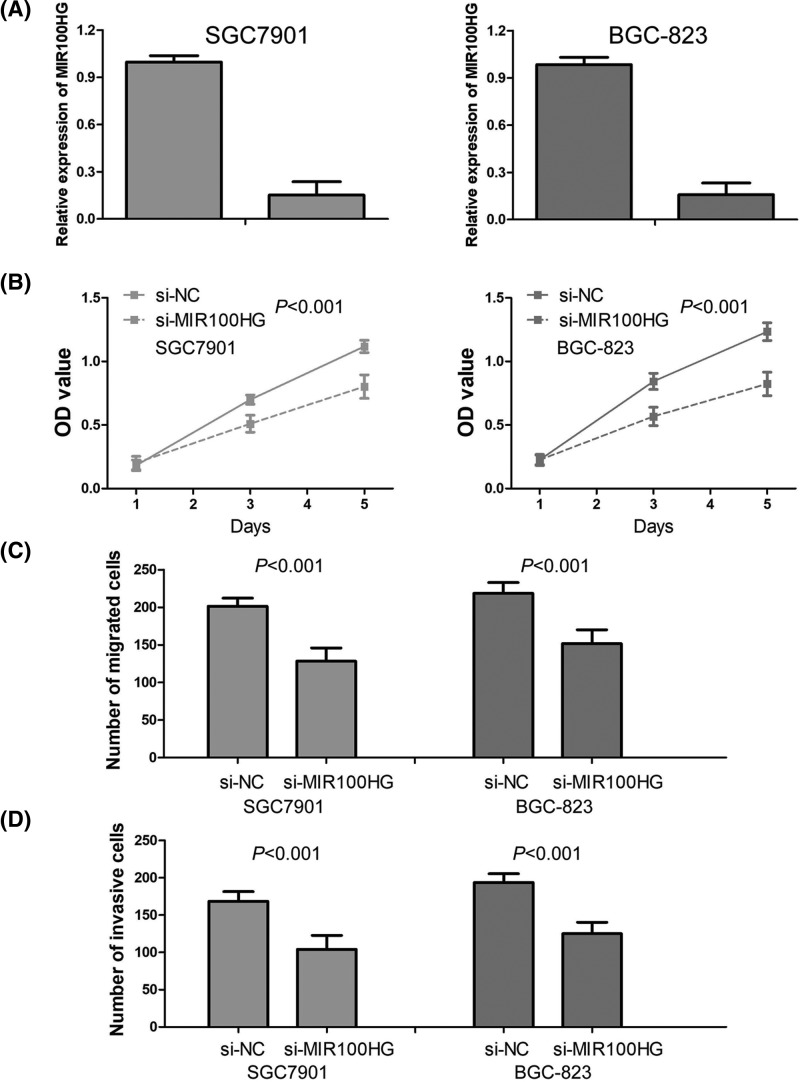
MIR100HG is associated with poor clinical outcome in gastric cancer (**A**) The transfection efficiency of si-MIR100HG was confirmed by qRT-PCR in SGC7901 and BGC-823 cell lines. (**B**) The effect of MIR100HG on the proliferation ability of gastric cancer cells *in vitro* was measured by CCK-8 assay. (**C**) The effect of MIR100HG on gastric cancer cell migration was detected by transwell migration assay. (**D**) The effect of MIR100HG on gastric cancer cell invasion was determined by transwell invasion assay.

We found that MIR100HG expression was relatively increased in SGC7901, BGC-823 cell lines amongst four gastric cancer cell lines (Figure 1B). Thus, SGC7901 and BGC-823 cell lines were chosen for loss-of-function experiments through si-MIR100HG transfection. The transfection efficiency of si-MIR100HG was confirmed by qRT-PCR in SGC7901 and BGC-823 cell lines (Figure 3A). To investigate the effect of MIR100HG on the proliferation ability of gastric cancer cells *in vitro*, CCK-8 assay was conducted and showed that down-regulation of MIR100HG expression obviously suppressed gastric cancer cell proliferation (*P*<0.001, Figure 3B). Additionally, transwell migration and invasion assays were conducted to measure the migration and invasion ability of gastric cancer cells after the transfection of si-MIR100HG. The results suggested that down-regulation of MIR100HG expression significantly reduced gastric cancer cell migration and invasion (*P*<0.001, Figure 3C,D).

## Discussion

Recent years, the role of MIR100HG in human cancer is gradually drawing the attention by oncologists. The MIR100HG expression was observed to be up-regulated or down-regulated in human cancer tissues depending on tumor types. In non-small-cell lung cancer, Yu et al. performed microarray gene expression analysis at two lung cancer microarray datasets and found MIR100HG expression was reduced in tumor tissues compared with normal lung tissues [26]. Moreover, Wieczorek et al. observed that MIR100HG was down-regulated in muscle invasive bladder cancer compared with the normal tissues [1]. On the contrary, high levels of MIR100HG expression were found in colorectal cancer [10], osteosarcoma, [18] and acute megakaryoblastic leukemia [7]. In colorectal cancer, Lu et al. found tumor tissues and cell lines exhibited MIR100HG overexpression [10]. Additionally, Su et al. suggested MIR100HG expression levels in osteosarcoma tissues and cell lines were elevated in comparison with corresponding para-tumor tissues and osteoblast cell lines, respectively [18]. Besides, Emmrich et al. reported MIR100HG are highly expressed in acute megakaryoblastic leukemia blasts [7]. However, the expression status of MIR100HG in gastric cancer was still unknown. In our study, we first found levels of MIR100HG expression were increased in gastric cancer cell lines and tissue samples compared with normal gastric epithelial cell line and adjacent normal gastric mucosa tissue samples, respectively.

The clinical value of MIR100HG in gastric cancer was further explored through analyzing the association between MIR100HG expression and clinicopathological features. We observed high MIR100HG expression was positively associated with clinical stage, tumor invasion, lymph node metastasis, and distant metastasis in gastric cancer patients. In colorectal cancer, Lu et al. suggested MIR100HG acted as a potential biomarker for predicting cetuximab resistance [10] Moreover, Shang et al. reported that MIR100HG overexpression was correlated with pelvic lymph node metastasis in early-stage patients with cervical cancer [16]. In breast cancer, Wang et al. found MIR100HG expression was higher in triple-negative breast cancer than other tumor types [22]. Su et al. also found MIR100HG overexpression was associated with large tumor size and advanced clinical stage in osteosarcoma cases [18].

The prognostic significance of MIR100HG expression has been investigated in breast cancer [22], bladder cancer [1], cervical cancer [16], osteosarcoma [18], and head and neck squamous cell carcinoma [24]. In breast cancer, Wang et al. revealed that patients with high MIR100HG expression had unfavorable prognosis than patients with low MIR100HG expression [22]. Although MIR100HG expression was suggested to be reduced in bladder cancer, high MIR100HG expression was associated with poor clinical outcome [1]. In early-stage cervical cancer patients, Shang et al. observed that MIR100HG expression had negative correlation with poor overall survival [16]. Besides, Su et al. showed osteosarcoma cases with high MIR100HG expression had poorer prognosis than those with low MIR100HG expression [18]. In head and neck squamous cell carcinoma patients, Wilkins et al. demonstrated that MIR100HG variant rs1816158 was associated with poor overall survival [24]. Up to now, the prognostic significance of MIR100HG expression in gastric cancer was still unclear. We first found MIR100HG expression was negative correlated with clinical outcome in gastric cancer patients from TCGA database or our study. Meanwhile, high MIR100HG expression served as an independent poor prognostic factor for gastric cancer patient’s overall survival.

There was no report about the biological function of MIR100HG expression in gastric cancer cells. We preliminarily investigated the effect of MIR100HG on gastric cancer cell proliferation, migration and invasion, and found down-regulation of MIR100HG expression inhibits cell proliferation, migration and invasion in gastric cancer. In addition, Wang et al. showed knocking down MIR100HG expression inhibited cell proliferation and arrested cell-cycle at G1 phase [22]. In osteosarcoma cells, inhibition of MIR100HG inhibited cell proliferation and cell cycle process, and enhanced cell apoptosis [18]. Besides, Emmrich et al. down-regulation of MIR100HG suppressed leukemic growth of acute megakaryoblastic leukemia cell lines and primary patient specimens [7].

In conclusion, MIR100HG is overexpressed in gastric cancer tissues and cell lines, and correlated with clinical progression and poor clinical outcome. Down-regulation of MIR100HG expression inhibits cell proliferation, migration and invasion in gastric cancer.

## Abbreviations

FBS: fetal bovine serum
qRT-PCR: quantitative real-time PCR
TCGA: The Cancer Genome Atlas
